# Public health nursing education viewed through the lens of superdiversity: a resource for global health

**DOI:** 10.1186/s12912-020-00411-3

**Published:** 2020-03-20

**Authors:** Cécile-Marie Dupin, Mélanie Pinon, Karine Jaggi, Celina Teixera, Aurèle Sagne, Noelia Delicado

**Affiliations:** 1 Geneva School of Health Sciences, HES-SO University of Applied Sciences and Arts Western Switzerland, Avenue de Champel 42, Geneva, Switzerland; 2 Faculty of Medical and Nursing Science, Aix-Marseille University, Faculté des Sciences Médicales et Paramédicales, 27 BVD Jean Moulin, 13005 Marseille, France

**Keywords:** Bologna process, Competencies, Diversity, Global Health, Nursing curricula

## Abstract

**Background:**

Nurses are increasingly confronted with the challenge of globalization and the acceleration of migratory flows. This reality affects the notion of culture and its influence on health-related behaviors. The state of health of the population in the Canton of Geneva, where there is a wide diversity of origins, is characterized by significant differences. The term “superdiversity” is used to describe the increasing complexity in ethnic diversity due to migration and social stratification. Nursing education in Geneva, influenced by the Bologna Process, appears appropriate for superdiverse contexts of care, with the development of dedicated competencies.

**Aim:**

This discussion paper aims to examine the academic curricula implemented in Geneva in the light of the concept of superdiversity.

**Main text:**

In Geneva, nursing education and curricula in public health are based on a competence framework for nursing care divided into 7 roles and educational tracks. Bachelor’s-level nurses know how to assess a care problem quickly and solve it effectively by setting relevant priorities, and do so based on evidence. The curricula aim to teach nurses to design population and individual interventions in their superdiverse context.

**Discussion:**

Education should enable students to develop their role as health promoters for the well-being of patients and communities, taking into account cultural complexity.

**Conclusions:**

Superdiverse contexts highlight the role of nurse educators in preparing future generations of public health nurses.

## Background

### From vocational to academic nursing programs

The nursing profession has experienced fundamental transitions and transformations over the past 50 years. Faced with the health care needs of the twenty-first century [7], it is difficult to see how nurses could develop essential skills (providing patient-centered care, working in interdisciplinary teams, improving the quality of continuous care, using information systems, and evidence-based practice) without the benefit of university education [21]. Amid the academic evolution of nursing in Europe, the Bologna Process aims to harmonize higher education by converging towards a common European system, in which different national systems share Bachelor’s, Master’s, and Doctorate degrees [29, 30, 34]. Historically, European higher education was reserved for the upper classes and had no place for schools of nursing. Previously, nurses were typically trained at the vocational level and obtained their education at institutions other than higher education entities [6]. Switzerland is one such European country, where nursing education has only recently begun to evolve from vocational education into undergraduate and graduate education, aided by the Bologna Process. Such transitions aim to facilitate the integration of scientific knowledge into clinical practice, an approach known as Evidence-Based Practice (EBP). The premise of EBP is that rather than focusing their practice solely on traditions and knowledge, professionals should seek information rooted in scientific development [19, 20]. Similarly, Evidence-Based Nursing (EBN) is a concept that embraces not only research results, but also all other sources of clinical knowledge (expertise, intuition, clinical insight, and perspicacity), as well as patient preferences, in order to make informed decisions [8].

### The public health perspective in nursing education

Public and community health share a common objective: improving the health status of individuals, collectively rather than individually [28]. Today, the increase in social and territorial health inequalities and lifestyle changes, and their consequences on the health status of populations, call for actions on environments, living environments, and the social determinants of health (SDH), achieved through intersectoral and participatory programs [28]. Action and research for population intervention planning must take into account SDH, which offers a wealth of factors to inform decision-making in health promotion. Research conducted in the field of nursing must contribute to the production of useful and usable knowledge [12, 13], particularly through intervention research, in order to evaluate existing strategies and contribute to improving the health of populations [25, 26]. In addition, empowerment strategies for communities targeted by health promotion actions must influence the conditions in which people are born, grow, live, work, and age [33].

Nursing education’s transition to higher education has made nurses better-prepared for their future roles, enabling them having a positive impact on the health of individuals and populations in terms of health promotion.

## Main text

### Globalization in public health: diversity and disparity

#### Global health

Study, research, and practice into global health places a priority on improving health and achieving equity in health for all people worldwide [17]. Global health emphasizes transnational health issues, determinants, and solutions; it involves many disciplines within and beyond the health sciences; it promotes interdisciplinary collaboration; and is a synthesis of population-based prevention with individual-level clinical care [17]. Diversity stemming from migration is one of Switzerland’s key characteristics, notably in Geneva Canton. In 2017, this urban canton was home to almost 500,000 people, making it the sixth-largest canton in Switzerland in terms of population – one that grew by 6.9% between 2012 and 2017), in no small part due to migration. In 2017, 62.5% of the population consisted of migrants, compared to 37.2% for Switzerland as a whole [31]. This diversity stemming from migratory movement is a source of enrichment, but also contributes to the existence of health, social, and economic disparities in Geneva Canton (see Table 1).

**Table 1:** Geneva Canton health data (Excerpt adapted from [31])

Health indicators	Migratory status	Geneva	Switzerland
		%	%
Proportion of individuals with a (very) good self-assessed state of health	Non-immigrant	84.7	86.7
	1st & 2nd gen. Immigrants	82.7	83.1
Proportion of individuals with serious limitations in activities usual for others	Non-immigrant	5.3	4.1
	1st & 2nd gen. Immigrants	5.6	4.3
Proportion of individuals with symptoms of depression	Non-immigrant	12.2	6.7
	1st & 2nd gen. Immigrants	13.5	11.8
Proportion of individuals with poor social support	Non-immigrant	9.7	7.9
	1st & 2nd gen. Immigrants	14.7	13.7
Individuals affected by pollution at work	Non-immigrant	42.5	47
	1st & 2nd gen. Immigrants	54.2	50.5
Proportion of individuals suffering from physical stress at work (painful or tiring postures, lifting or moving heavy loads, lifting or moving persons)	Non-immigrant	12.2	13.3
	1st & 2nd gen. Immigrants	19.5	22.1
Proportion of individuals suffering from emotional fatigue at work (burnout)	Non-immigrant	20.6	17.6
	1st & 2nd gen. Immigrants	24.3	21.9

### The concept of superdiversity

The scientific literature explores the cultural problems encountered by these populations, their state of health, and their means of access to care [27]. The concept of “superdiversity” relates to the differential convergence of factors surrounding patterns of immigration arising from the complex interplays between newcomers’ experiences, opportunities, constraints, and trajectories, and the wider set of social and economic relations within the places where they reside. Such factors include: country of origin (comprising a variety of possible subset traits such as ethnicity, language[s], religious tradition, regional and local identities, cultural values and practices), migration channel (often related to highly gendered flows and specific social networks), legal status (determining entitlement to rights), migrants’ human capital (particularly educational background), access to employment (which may or may not be available to immigrants), locality (related especially to material conditions, but also the nature and extent of other immigrant and ethnic minority presence), transnationalism (emphasizing how migrants’ lives are lived with significant reference to places and peoples elsewhere) and the frequently uneven responses by local authorities, service providers, and local residents (which often tend to function by way of assumptions based on previous experiences with migrants and ethnic minorities) [32, 35].

Superdiversity offers a fresh and innovative way of understanding local levels of disparity nested in the challenges of contemporary global health, and appears to be a promising lens through which to analyze public health nursing education.

### The need for a fresh perspective on disparity and diversity in 2020

While diversity is not a new concept in nursing, further integration of cultural diversity in educational systems and curriculum design for nursing education can positively influence how future nurses integrate the characteristics of superdiverse contexts in their practices.

### The new Swiss community health education system

Since the promulgation of the Bologna Process, a number of different strategies have been adopted by individual countries, the European Union, governments, and professions in order to achieve the outcomes established by the Bologna Declaration. At country level, new policies to change the educational framework, or create the conditions for a new one, have been determined by individual governments responsible for higher education systems, individual universities, and professional associations and networks [22]. In Switzerland, the HES-SO (*Hautes Ecoles de Santé Romande*, western Switzerland’s applied science university network) provides an education that prepares students for professional activities requiring the application of scientific knowledge and methods, for the first cycle of studies: the Bachelor’s degree corresponds to 180 credits in the European Credits Transfer System (ECTS). With nine different courses and 3814 students, the HES-SO’s Health Department is the largest health university in Switzerland and the only one to offer the entire University of Applied Sciences (UAS) syllabus for health. For Geneva, the Best Practice KFH [1] provides a systematic list of competences targeted by the Bachelor’s degree in nursing. The competences covered by the Nursing Framework Curriculum express the knowledge and know-how that a specialist must possess to accomplish his or her main tasks. This definition of “competence” uses the terminology of the Copenhagen process (an EU project for the creation of a European vocational training area), which provides a framework for competence models. In this terminology, a “competence” refers to the ability to apply knowledge, know-how and interpersonal skills in a regular or new job. This ability is defined in terms of purpose, autonomy, sense of initiative, responsibilities, relational context, resource use, and profile requirements for a person with a Bachelor’s degree in nursing. Each competence has four elements: (1) Cognitive competence, which involves the use of theories/concepts and tacit knowledge gained through experience; (2) Functional competence, which implies a mastery of the skills and know-how necessary to perform a practical function; (3) Personal competence, which implies the ability to know how to behave in a given situation or in a specific professional situation; (4) Ethical competence, which implies reference to certain personal and social values.

Professionals from a UAS background are called upon to deal with problematic, complex, and hard-to-predict situations. Their professional interventions call simultaneously on specialized knowledge and varied methods of action for interventions that are inherently singular, since they are intended for human beings [10]. Bachelor’s nurses mobilize their skills, knowledge and professional attitude to provide supportive, preventive, therapeutic, palliative, and educational care oriented towards the person, family, or community. Bachelor’s-level nurses know how to quickly grasp a care problem and solve it effectively by setting relevant priorities, and do so based on evidence, whilst observing professional ethics [11].

The Swiss community nursing education aims to educate nurses in critical thinking as well as enabling them to act with the highest degree of professionalism.

### Future challenges for public health nursing education

Information and Communications Technology (ICT) plays a role in nursing student education, improving practices and adjusting them more appropriately to the challenges faced by professionals. However, for nursing, rather than investing in more technology and education, institutions and departments should aim to give more recognition, and respond better, to academics’ specific discipline-related needs, so as to reflect the practical realities of nursing education. This constitutes a challenge to traditional teaching models [23].

The Geneva context, characterized in particular by growing health inequalities and diversity, requires dedicated education about how to act on the environment and SDH, intersectoral (transversal) actions, and design participatory programs [14]. This relates to the advancement of knowledge about promising modes of action, SDH, and the development of strategies for their evaluation [9]. Health literacy refers to the individual’s ability to obtain, understand, and use the information required to take the appropriate decisions to remain in good health [16]. The concept was originally confined to individuals’ ability to read and understand written information, but has now been extended to many other factors affecting an individual’s ability to have access to, understand, and use information about health and health services [3]. In Geneva, amid high levels of migration, health literacy levels may reflect the diverse makeup of the Canton’s population [2]. Public health interventions may even increase social health inequities, in line with the “inverse prevention law”, which states that those in most need of preventive interventions are the least likely to receive them, whereas interventions should be of greater benefit to disadvantaged groups [18].

Public health nursing education faces a twofold challenge: educating nurses using appropriate teaching strategies in view of their professional specifics, and designing population and individual interventions for superdiverse contexts.

### Superdiversity and health-promoting nursing

The curricula provided by the HES-SO Health courses is relevant to society because it addresses the present and future health needs of the population and is adapted to the Swiss health system [11] (15). In Geneva, nursing education is based on a competence framework for nursing care, divided into 7 roles and tracks: content derived from scientific knowledge (disciplinary and contributory): nursing science, health sciences, human sciences, research (processes and methods); professional content: methods, approaches and tools of the profession, clinical skills; practical clinical periods: 6 periods of practical education; and a Bachelor’s dissertation that contributes to the integration of research results into professional practice. Community health education should enable students to become health promoters – and ensure that as health promoters, nurses use their expertise and influence to promote the health and well-being of patients and communities. The dimensions of the role of health promoter (or at least its significant aspects) include: health promotion, health education, prevention, and quality of life (individual-group-community). Today, the holistic nature of community practice and the diversity of practice learning environments provide students with opportunities to develop a range of skills for contemporary community nursing [4].

Academic curricula must enable nurses to meet these challenges and play this role to the full, as is already acknowledged in various countries, developing 1) public health/community health competences, 2) research competences, and 3) clinical nursing competences [5].

## Conclusions

Superdiversity demonstrates that cultural change does not occur overnight: it occurs over years and generations. Cross-cultural values are needed in a cross-cultural world, and nurse educators will need to lead the way in preparing future generations of international nurses [6]. Cross-cultural research among nurse educators is limited [15], but important: nurse educators are on the front line when it comes to educating the next generation of nurses [24].

## Data Availability

Data sharing is not applicable to this article as no datasets were generated or analyzed during the current study.

## Abbreviations

EBN: Evidence-Based Nursing
EBP: Evidence-Based Practice
ECTS: European Credits Transfer System
ICT: Information and Communications Technology
SDH: Social Determinants of Health
UAS: University of Applied Sciences
